# Adolescents and alcohol: an explorative audience segmentation analysis

**DOI:** 10.1186/1471-2458-12-742

**Published:** 2012-09-05

**Authors:** Jolanda Mathijssen, Meriam Janssen, Marja van Bon-Martens, Ien van de Goor

**Affiliations:** 1 Tranzo Department, Academic Collaborative Centre for Public Health Brabant, Tilburg University, Post Office Box 90153 5000, Tilburg, LE, The Netherlands; 2 Department of Health Promotion, Regional Health Service, Hart voor Brabant, 's-Hertogenbosch, The Netherlands

**Keywords:** Audience segmentation, Adolescents, Alcohol use

## Abstract

**Background:**

So far, audience segmentation of adolescents with respect to alcohol has been carried out mainly on the basis of socio-demographic characteristics. In this study we examined whether it is possible to segment adolescents according to their values and attitudes towards alcohol to use as guidance for prevention programmes.

**Methods:**

A random sample of 7,000 adolescents aged 12 to 18 was drawn from the Municipal Basic Administration (MBA) of 29 Local Authorities in the province North-Brabant in the Netherlands. By means of an online questionnaire data were gathered on values and attitudes towards alcohol, alcohol consumption and socio-demographic characteristics.

**Results:**

We were able to distinguish a total of five segments on the basis of five attitude factors. Moreover, the five segments also differed in drinking behavior independently of socio-demographic variables.

**Conclusions:**

Our investigation was a first step in the search for possibilities of segmenting by factors other than socio-demographic characteristics. Further research is necessary in order to understand these results for alcohol prevention policy in concrete terms.

## Background

Dutch adolescents often start drinking alcohol at an early age. The life-time prevalence for drinking alcohol is 56% for twelve year olds and 93% for sixteen year olds. Also, 16% of twelve year olds and 78% of sixteen year olds drink alcohol regularly. In comparison with other young people in Europe, Dutch adolescents drink more frequently and are more likely to be binge drinkers (episodic excessive alcohol consumption, defined as drinking 5 glasses or more on a single occasion in the last four weeks) [1].

Despite a sharp decline in the excessive consumption of alcohol (6 or more glasses at least once a week for the last 6 months) among adolescents in the Netherlands, the alcohol consumption is still high [2]. Data from the Regional Health Services (RHS) in the province of North Brabant [3] also show this. Although the number of young people who regularly consume alcohol (at least once in the past 4 weeks) declined from 54% in 2003 to 44% in 2007, 28% of the 12 to 17 year olds in the area of the RHS “Hart voor Brabant” can be identified as binge drinkers. Moreover, 25% of the under 16s are regular drinkers, and 13% are even binge drinkers.

Alcohol consumption by adolescents under 16 causes severe health risks. Firstly, young people's brains are particularly vulnerable because the brain is still developing during their teenage years. Alcohol can damage parts of the brain, affecting behavior and the ability to learn and remember [4]. Secondly, there is a link between alcohol consumption and violent and aggressive behavior [5-7] and violence-related injuries. Thirdly, young people run a greater risk of alcohol poisoning when they drink a large amount of alcohol in a short period of time [8]. Finally, the earlier the onset of drinking, the greater is the chance of excessive consumption and addiction in later life [9-11].

The policy of the Dutch Ministry of Health is aimed at preventing alcohol consumption among adolescents younger than 16, and at reducing harmful and excessive drinking among 16–24 years old young adults [12]. Local Authorities are responsible for the implementation of national alcohol policy at a local level. RHSs and regional organizations for the care and treatment of addicts carry out prevention activities at a regional and local level, often commissioned by Local Authorities.

Current policies and interventions are mainly directed at settings such as schools and sports clubs. However, it is unlikely that this approach will have sufficient impact on adolescents, because the groups in these settings are heterogeneous. Adolescents differ in their drinking habits and have different attitudes towards alcohol. This means that one intervention reaches only a part of all adolescents, and doesn’t reach other adolescents, with a different drinking habit or a different attitude.

Market research has revealed the importance and effectiveness of tailoring messages and incentives to meet the needs of different population segments. Not every individual is a potential consumer of a given product, idea, or service; so tailoring messages to specific groups will be more effective than broadcasting the same message to everyone [13,14].

Audience segmentation is a method for dividing a large and heterogeneous population into separate, relatively homogeneous segments on the basis of shared characteristics known or presumed to be associated with a given outcome of interest [15].

Audience segmentation is fairly common in the field of public health. However, such segmentation is usually based on socioeconomic and demographic variables, such as age, ethnicity, gender, education and income. Unfortunately, demographic segmentation alone may be of limited use for constructing meaningful messages [16]. While psychographic and lifestyle analyses have long been standard practice in business marketing, their use in public health communication efforts is still much less common [16]. Since health messages can be fine-tuned to the differences in lifestyle such as attitudes and values, segments based on aspects of lifestyle are expected to be more useful for health communication strategies [14,16]. We assume that attitudes, values, and motives in relation to alcohol consumption among adolescents will vary, and may therefore offer a better starting point for segmentation than socio-demographic characteristics alone. For example, previous research has shown that motives for drinking give rise to a substantial part of the variance in alcohol consumption [17,18]. Moreover, personality traits, such as sensation seeking, are associated with quantity and frequency of alcohol use [19].

Despite the promising characteristics of audience segmentation based on lifestyle aspects, it has never been used in the Netherlands in relation to the prevention of alcohol consumption. That is why the RHS “Hart voor Brabant”, in cooperation with market research office Motivaction®, conducted a study to find out whether it is possible to identify different segments on the basis of the motives, attitudes, and values of adolescents towards alcohol. The first results of this study were already published in a Dutch article [20].

## Methods

### The sample

A random sample of 7,000 young people aged 12 to 18 was drawn from the Municipal Basic Administration (MBA) of the 29 municipalities in the area of the RHS “Hart voor Brabant”. The personal data of each member of the Dutch population is held in the MBA. The survey was approved by the board of directors of the RHS, and exempted from ethical approval. According to the Dutch Medical Research Involving Human Subjects Act (WMO) these surveys were exempted from ethics approval because they did not meet the criterion that people are subjected to (invasive or bothersome) procedures or are required to follow rules of behavior. Adolescents aged 16 or over received a letter containing an internet link to a questionnaire and a password. For adolescents under 16 years of age, this letter was sent to the parents with the request to allow their son/daughter to fill in the questionnaire. In order to increase the response rate, two reminders were sent to non-respondents, respectively two and four weeks after the letter of invitation. As an incentive, one in ten young people who filled in the questionnaire received a € 10 cinema coupon.

### Questionnaire

In composing the questionnaire we used questions from the local and national youth health monitor. The questions concerned demographic characteristics, alcohol consumption, smoking habits, and leisure activities [21]. Since there was no validated questionnaire available to study the attitude of adolescents to alcohol, we developed in total 56 propositions concerning general values and attitudes and specific values and attitudes relating to alcohol. These propositions were based on a brief literature study, a workshop with experts, and focus groups with adolescents from the region. Epidemiologists, health policy officials, and addiction experts participated in the workshop with experts. Two focus group interviews were held among young people with the aim of obtaining greater insight into the attitudes and experiences of young people in relation to alcohol. To ensure that there was sufficient spread in alcohol consumption levels, gender, age, and educational level within the focus groups, the participants filled in a short questionnaire prior to the interviews. One focus group consisted of four girls and three boys, varying in age from 13 to 16, attending a representative range of secondary school types. The alcohol consumption varied from never to now-and-then. The other focus group consisted of three girls and three boys. This group also varied in age and educational level. However, the level of alcohol consumption in this group was higher than in the other focus group (varied from now-and-then to regularly).

### Final workshop

After analyzing the data from the questionnaire a final workshop was organized. Twelve representatives of Local Authorities, of the Netherlands Youth Institute, of the Netherlands Institute for Social Research, and of the RHS “Hart voor Brabant” took part in this workshop. As the first step during this meeting, the results of the analysis of the questionnaire answers were presented. Then, using a range of working methods (including a plenary discussion and assignments in subgroups), the results were further explored with the aim of understanding the significance of the segments and of working them out in more detail.

### Statistical analysis

Data were analyzed using SPSS 17.0 for windows (factor analysis, ANOVA, Χ^2^-analyses, and multiple logistic regression analyses) and Latent Gold (latent class analysis).

Factor analysis using principal components with oblimin rotation was conducted. The factors retained were based on the following rules: eigenvalues greater than or equal to 1 or factors above the break in the scree plot and a minimum of 0.40 for factor loadings.

A latent class analysis was then carried out with the factors found in the factor analysis, using the software package Latent Gold [22]. In this analysis, a cluster solution was sought according to the following criteria. First, the statistical fit of the latent class model with the data was considered (p > 0.05, the null hypothesis is that there is no significant deviation between the predicted data and the observed data). Then the most economical model was chosen from those that fitted. That is the model for which the Bayesian Information Criterion (BIC value) is the lowest. This value is higher for more complex, and therefore less economical, models.

Analyses of variance (ANOVA) were used to examine differences between the found segments and factor scores and age. With chi-square analyses we investigated whether the segments differed in categorical socio-demographic variables (sex, ethnic background and urbanization) and alcohol consumption (recent alcohol use and binge drinking).

Multiple logistic regression analyses were used to examine whether the segments were significantly associated with alcohol consumption, independent of socio-demographic characteristics. The data were entered in two blocks. The first block consisted of socio-demographic variables (age, sex, urbanization, and ethnicity). In the second block, the segment variable was entered.

## Results

A total of 3,230 12 to 18-year-olds completed the questionnaire. This is a response of 46%. The mean age of the respondents was 14;11 and 49% of them was female. More than half of them lived in a (highly) urbanized area (56%) and 16% was from ethnic minorities. These percentages are comparable to the population of 12–18 years old living in the working area of the RHS, respectively 15;0 years old, 49% girls, 56% (highly) urbanized area and 17% ethnic minorities.

### Factor analysis

A first exploratory factor analysis, with oblimin rotation, based on the 56 propositions concerning alcohol and other matters that young people value important (such as parents, school, classmates, and sports) produced 14 factors that satisfied the criterion eigenvalue > 1. On studying the eigenvalues per factor on a scree plot, specifically between the 10th and 11th factor, a relatively large fall in the eigenvalue was to be seen. Beyond the 10th factor, the eigenvalue gradually decreased further. For this reason, a new analysis was carried out in which the number of factors was fixed at 10. This analysis eventually produced seven factors with sufficient reliability (Cronbach’s Alpha >0.60). Of the remaining seven factors, two were excluded on content grounds. One of these consisted of items that dealt with being sufficiently well informed and warned about alcohol. This factor is ambiguous, in the sense that it is unclear whether respondents who agree with this consider themselves sufficiently well-informed because of the amount of information provided, or because they do not feel the need for information. Therefore we found this factor unsuitable as a basis for the segmentation. The other factor was represented by a block of four items about rules that ought to be imposed by parents. These items had been placed together in the questionnaire. Because it was not possible to establish whether the consistency of the replies to these questions was a result of the composition of the questionnaire, it was decided to exclude this factor as well, as a basis for the segmentation. The five remaining factors, which we found suitable for our segmentation analysis, together accounted for 53% of the variance.

These factors are ‘aversion to intoxication’ (α = 0.83), ‘alcohol is the norm’ (α = 0.61), ‘need for approval’ (α = 0.67), ‘hedonistic associations’ (α = 0.75) and ‘lack of interest in alcohol’ (α = 0.86). The items belonging to the factors are described below. For the factor loadings see Additional file 1.

Items per factor

Aversion to intoxication

I would be embarrassed if one of my friends got drunk

My friends would be embarrassed if I got drunk

I would be embarrassed if I got drunk myself

I would find it amusing if one of my friends got drunk *(recoded)*

My parents would be embarrassed if I got drunk

People are more fun when they've been drinking *(recoded)*

Stronger action should be taken against alcohol misuse

People become annoying when they've been drinking

Alcohol as norm

I can imagine that you don't want to be seen with a soft drink when everyone else is drinking alcohol

It’s weird if an adult never drinks alcohol

Drinking alcohol is more fun when it's not allowed

Since it's legal to buy alcohol once you are 16, it must be less damaging from that age

I think it's exciting to be drunk

For me it's important not to be different from other people

Alcohol is more for boys than for girls

Need for approval

The opinion of my parents is important to me

My parents take my opinion seriously

It's important to me that my friends have a good opinion of me

It seems only natural to me to keep to my parents’ rules

I learn from my mistakes

Hedonistic associations

Alcohol makes me think of the weekend

Alcohol makes me think of relaxing

Alcohol makes me think of having fun

Alcohol makes me think of a drink with a meal

Alcohol makes me think of letting go

Alcohol makes me think of adulthood

Lack of interest in alcohol

Alcohol makes me think: Don't like the taste

Alcohol makes me think: Not for me

### Latent class analysis

**Figure 1:**
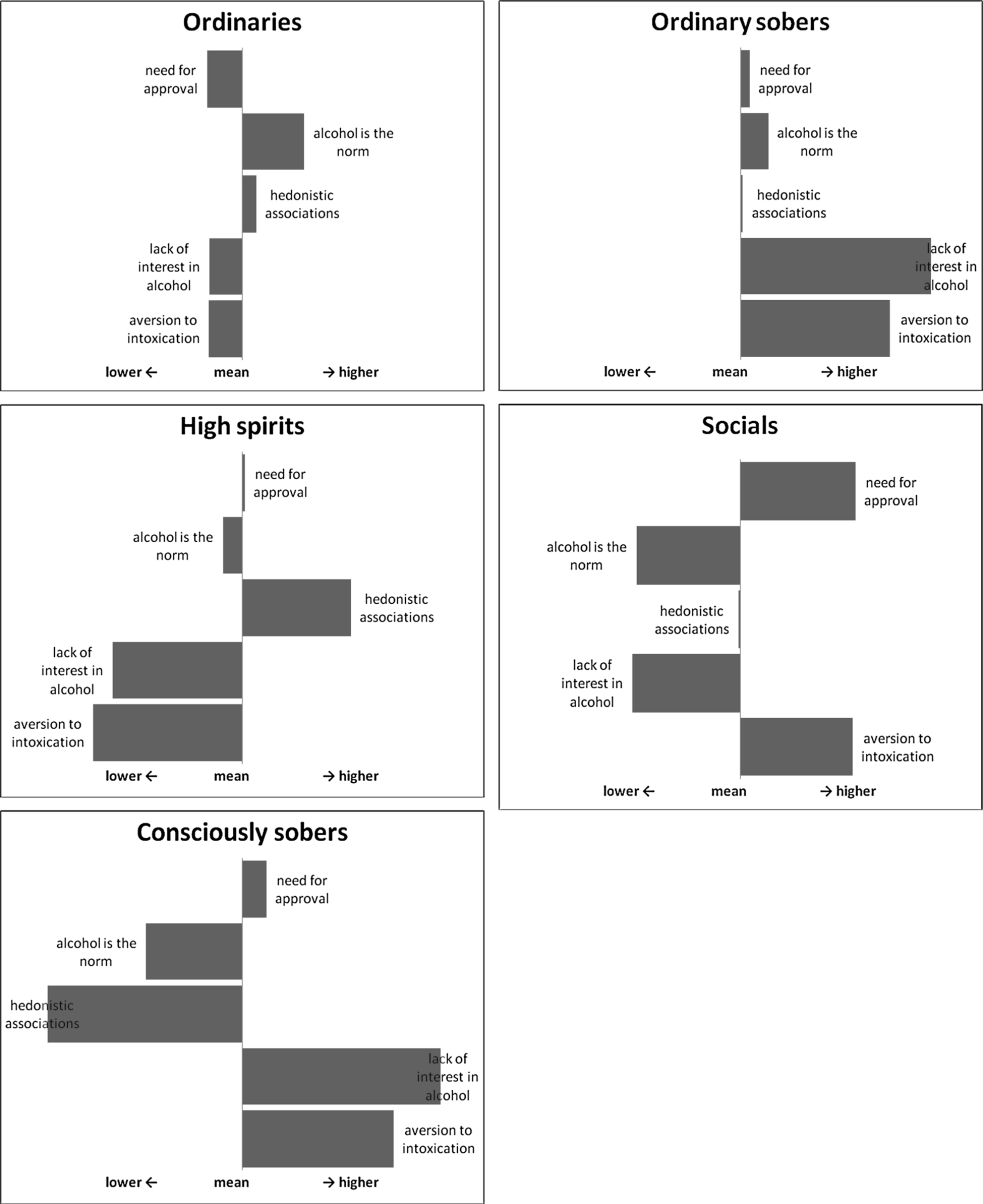
Standardized factor scores for the five segments.

In Table 1, we compare models with one to ten latent classes; we present the BIC and p-value for each model. The BIC statistic for the five class model was slightly lower than for the other models. Moreover, the five-class solution was the first model with p > 0.05. These findings indicated that the five-class solution was the best fit (BIC = 34906, p = 0.51). These five clusters were distinguished from each other by differences in the scores on the five attitude factors. In a workshop with experts we assigned (Dutch) names to them. In this English article we refer to the clusters as: ‘ordinaries’ (42%), ‘high spirits’ (22%), ‘consciously sobers’ (17%), ‘ordinary sobers’ (11%), and ‘socials’ (8%) (see Figure 1). The scores in Figure 1 have been standardised (μ = 0 and SD =1). Table 2 shows the average scores on the five attitude factors for each of the clusters (the segments). The figure and the table show clearly that the segments differ from each other in factor scores. For example, ‘high spirits’ have much less aversion to intoxication, greater interest in alcohol, and more hedonistic associations with alcohol than the other segments. The ‘ordinaries’ have less aversion to intoxication than the ‘consciously sobers’, the ‘ordinary sobers’, and the ‘socials’. The ‘ordinaries’ have the highest score on ‘alcohol is the norm’ and the lowest on ‘need for approval’. By contrast, ‘socials’ have the lowest score on ‘alcohol is the norm’ and the highest on ‘need for approval’. Finally, the ‘consciously sobers’ and the ‘ordinary sobers’ differ from each other on ‘hedonistic associations’ and ‘alcohol is the norm’. On both factors, the ‘ordinary sobers’ score higher than the ‘consciously sobers’.

**Table 1:** Model fit statistics for ten latent class models

	**BIC(LL)**	**p-value**
1-Cluster	37009,0	0,00
2-Cluster	35470,9	0,00
3-Cluster	35112,4	0,00
4-Cluster	35021,0	0,00
**5-Cluster**	**34906,3**	**0,52**
6-Cluster	34922,2	0,75
7-Cluster	34933,9	0,92
8-Cluster	34945,8	0,98
9-Cluster	34970,2	0,99
10-Cluster	34999,5	1,00

BIC = Bayesian Information Criteria.

LL = Log Likelihood.

**Table 2:** Mean scores (SD) on the 5 factors for the 5 segments

	**Aversion to intoxication**	**Lack of interest in alcohol**	**Hedonistic associations**	**Alcohol is the norm**	**Need for approval**
Ordinaries	2.4 (0.6) ^h c os s^	1.7 (0.7) ^h c os s^	2.6 (0.6) ^h c^	2.2 (0.6) ^h c os s^	3.3 (0.6) ^h c os s^
High spirits	1.7 (0.6) ^o c os s^	1.0 (0.0) ^o c os s^	3.2 (0.5) ^o c os s^	1.8 (0.7) ^o c os s^	3.5 (0.6) ^o s^
Consciously sobers	3.5 (0.6) ^o h s^	3.5 (0.8) ^o h s^	1.6 (0.6) ^o h os s^	1.8 (0.5) ^o h os^	3.6 (0.5) ^o s^
Ordinary sobers	3.5 (0.5)^o h s^	3.4 (0.7) ^o h s^	2.6 (0.7) ^h c^	2.0 (0.7) ^o h c s^	3.6 (0.6) ^o s^
Socials	3.3 (0.5) ^o h c os^	1.2 (0.4) ^o h c os^	2.6 (0.8) ^h c^	1.4 (0.5) ^o h c os^	3.9 (0.2) ^o h c os^

^o^ = Ordinaries, ^h^ = High spirits , ^c^ = Consciously sobers, ^os^ = Ordinary sobers, ^s^ = Socials (p ≤ 0.01).

### Background variables

A significant difference was found for sex (Χ^2^ = 13.9, df = 4, p ≤ .01). Girls were slightly overrepresented in the segments ‘ordinary sobers’ (55%), ‘socials’ (56%) and underrepresented in the segment 'high spirits’ (45%). Moreover, a difference relating to age was found (F = 146.1, df = 4, p ≤ .01). On average, the ‘high spirits’ were older (15 years; 11 months) than the ‘ordinaries’ (14;10), ‘consciously sobers’ (14;01) and ‘ordinary sobers’ (13;11). On average, the ‘socials’ (15;07) were older than the ‘ordinaries’, ‘consciously sobers’, and ‘ordinary sobers’. On average, the ‘ordinaries’ were older than the ‘consciously sobers’ and the ‘ordinary sobers’. Table 3 shows the distribution of ages within the different segments.

**Table 3:** Age distribution (percentage) by segment

	**12**	**13**	**14**	**15**	**16**	**17**	**18**	**Total**
Ordinaries	8	17	22	20	13	11	9	100
High spirits	2	3	10	22	25	24	14	100
Consciously sobers	18	25	21	14	10	9	3	100
Ordinary sobers	20	28	21	15	6	5	5	100
Socials	4	10	14	16	18	23	15	100

Moreover, we found a statistically significant difference for ethnic background (Χ^2^ = 258.3, df = 4, p ≤ .01). In total, 16% of the youngsters was from an ethnic minority. The segments ‘consciously sobers’ (36%) and ‘ordinary sobers’ (26%) showed an overrepresentation of ethnic minorities, while ethnic minorities were under-represented in the segments 'ordinaries' (12%), ‘high spirits’ (6%) and ‘socials' (9%).

Finally, the ‘ordinaries’ lived less often and the ‘consciously sobers’ lived more often in an (highly) urbanized area (Χ^2^ = 18.7, df = 4, p ≤ .01), respectively 53% and 62%.

### Alcohol consumption

We then investigated, with a chi-square test, whether the five segments differed in terms of recent alcohol consumption (alcohol consumed in the four weeks prior to the completion of the questionnaire) and binge drinking (the consumption of five or more glasses of alcohol on a single occasion in the preceding four weeks). Both differences were statistically significant (Χ^2^ = 1154.2, df = 4, p ≤ .01 for alcohol consumption and Χ^2^ = 1009.8, df = 4, p ≤ .01 for binge drinking).

**Figure 2:**
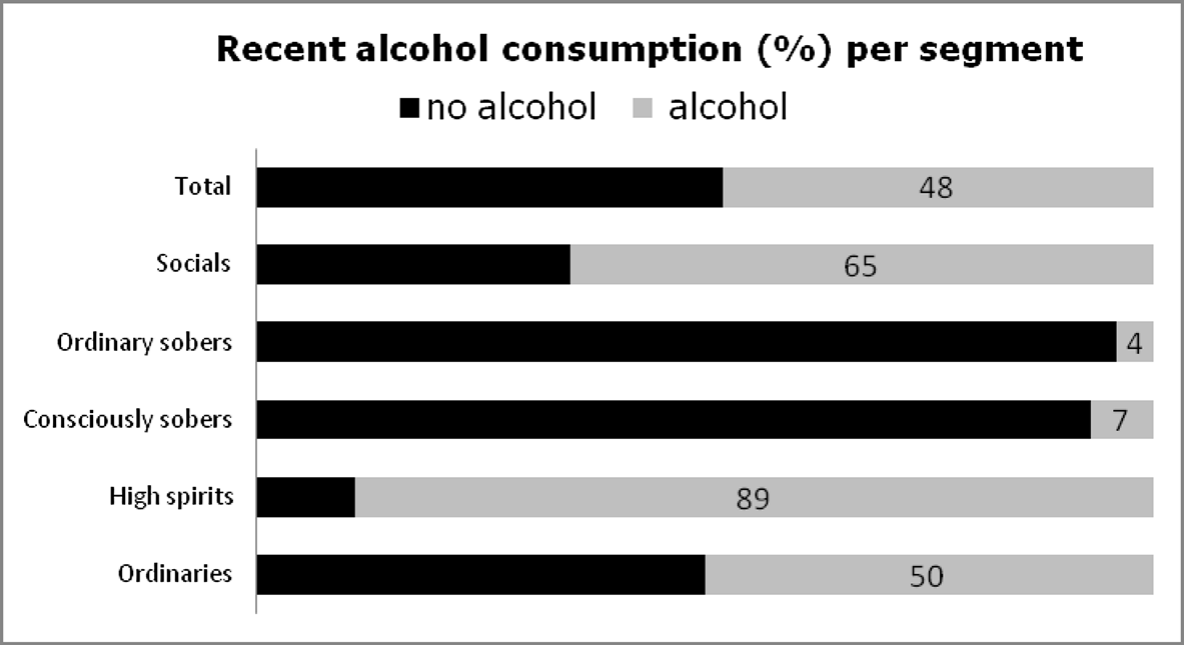
Recent alcohol consumption (%) per segment.

Figure 2 shows that, on average, 48% of the young people had recently drunk alcohol. This percentage was significantly higher for the ‘high spirits’ (89%) and the ‘socials’ (65%), and significantly lower for the ‘consciously sobers’ (7%) and ‘ordinary sobers’ (4%).

**Figure 3:**
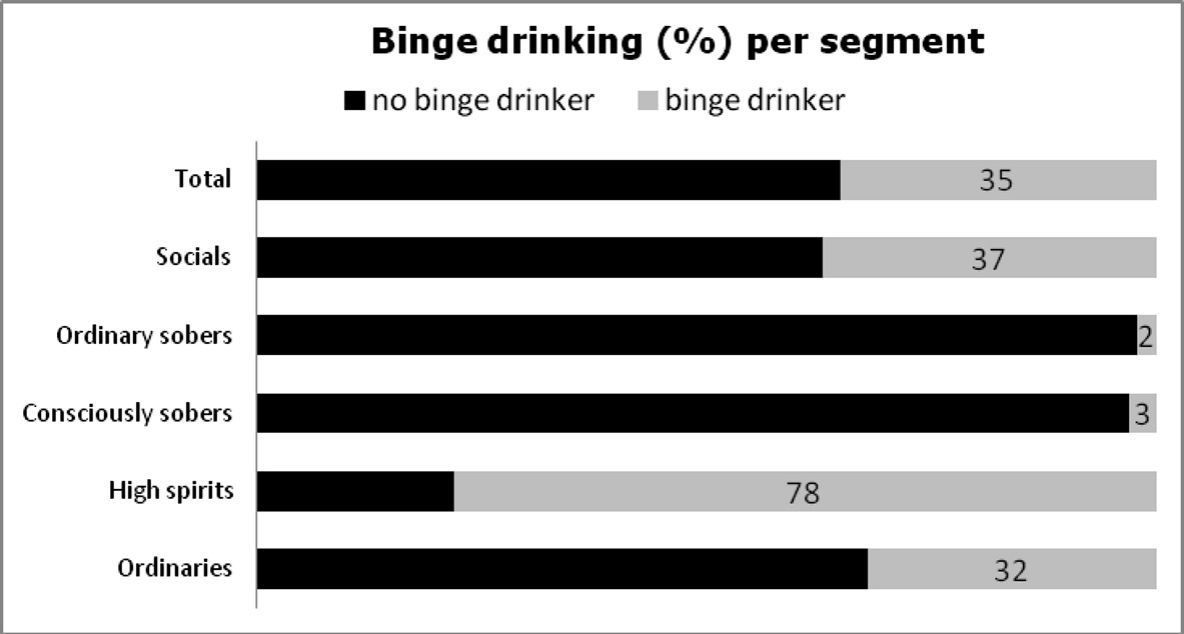
Binge drinking (%) per segment.

Of the adolescents, 35% can be classified as ‘binge drinkers’ (see Figure 3). This percentage is significantly higher for the ‘high spirits’ (78%), and significantly lower for the ‘consciously sobers’ (3%) and the ‘ordinary sobers’ (2%).

To examine whether the segments were significantly associated with alcohol consumption, independent of socio-demographic characteristics, we conducted multiple logistic regression analyses. For both recent alcohol consumption and binge drinking we ran two models; the first with only socio-demographic variables (age, sex, ethnicity, and urbanization) and the second one with the addition of the segment variable. As can be seen in Table 4 the segmentation variable was significantly associated with both recent alcohol consumption and binge drinking independent of socio-demographic variables. The Nagelkerke’s R^2^gives an approximation of the level of explained variance. In both cases the Nagelkerke’s R^2^ was higher for the second model with the segmentation variable.

**Table 4:** Association of the segments with recent alcohol consumption and binge drinking, adjusting for socio-demographic characteristics

	**Recent alcohol consumption**		**Binge drinking**	
	**Model 1**	**Model 2**	**Model 1**	**Model 2**
Age	2.23**	2.07**	1.98**	1.80**
Male	Ref	Ref	Ref	Ref
Female	1.19*	1.45**	0.85	0.95
(Highly) urbanized area	Ref	Ref	Ref	Ref
Non or little urbanized area	1.18	1.12	1.37**	1.38**
Native	Ref	Ref	Ref	Ref
Ethnic minority	0.21**	0.38**	0.35**	0.70*
Ordinaries		Ref		Ref
High Spirits		5.28**		5.24**
Consciously sobers		0.08**		0.08**
Ordinary sobers		0.04**		0.05**
Socials		1.10		0.81
Nagelkerk R^2^	0.42	0.61	0.33	0.53

### Final workshop

In the concluding workshop, the meaning of the segments was interpreted, the segments were described in more detail, and possible policy measures per segment were defined. This interpretation took place on the basis of a description of the segments with the help of questionnaire items, mood boards, and discussions among the participants. The ‘high spirits’ were typified as very direct, extrovert, sensation seekers, and focused on status. The ‘socials’ were described as sensible and purposeful. They also like to think things through, like to give their opinion, and have a good relationship with their parents. ‘Ordinaries’ were described as concerned about status, tractable, and focused on the familiar. ‘Consciously sobers’ were characterized as cautious, unadventurous, family-oriented, socially involved, and not particularly concerned about status. The ‘ordinary sobers’ were very similar to the ‘consciously sobers’ in terms of their value attitudes; cautious, unadventurous, family-oriented, and socially involved.

Finally, for each segment, possible approaches for policy and interventions were suggested. According to the participants of the workshop ‘high spirits’ must not be approached too didactically. However it is important to work on raising their awareness and the consequences of excessive alcohol consumption should be demonstrated. Finally, for this segment, possibly strict rules should be applied (e.g. alcohol-free school parties). ‘Socials’ seem to set their own limits, and have a sense of responsibility. Because the results suggest that ‘socials’ have a good relationship with their parents, the parents can play an important role in setting rules and reaching agreements with their children. The ‘ordinaries’ will see the ‘high spirits’ and ‘socials’ as strong role models. So, to make ‘ordinaries’ realize that not every young person drinks, it is important to increase the visibility of ‘consciously sobers’ and ‘ordinary sobers’. The ‘consciously sobers’ are non-drinkers on principle. It is therefore important for them that they should not feel isolated. For the ‘ordinary sobers’ it seems to be important that they should be stimulated to continue to say ‘no’.

## Discussion

In spite of the need within the field of health promotion for methods for dividing heterogeneous groups into more homogeneous subject-specific segments, there has generally been little progress beyond a division on socio-demographic characteristics. This investigation was a first step in the search for possibilities of segmenting by other than socio-demographic characteristics. The results of this investigation demonstrate that it is possible to group adolescents into five different and more homogeneous segments concerning values, attitudes, and motives in relation to alcohol. It is important to recognize that the names we gave to the segments are just labels, used to give a feeling for the characteristics of the five clusters. They are not descriptive names, and are not intended to represent the alcohol consumption of individual members of the clusters.

Besides the differences in values, attitudes, and motives, the segments also differed in drinking behavior. As an extension to the results presented in our Dutch article [20] we now studied whether alcohol specific segmentation was significantly associated with alcohol consumption independent of socio-demographic characteristics. These found differences for the segments remained even after controlling for age, sex, ethnicity and degree of urbanization.

Nevertheless, it is important to recognize that attitudes might change over time. A large proportion of the 12 and 13 year olds belong to the segments ‘ordinary sobers’ and ‘consciously sobers’. There is a possibility that, in one or two years, some members of the group ‘ordinary sobers’, and perhaps even of the ‘consciously sobers’, will move to one of the groups ‘high spirits’, ‘ordinaries’, or ‘socials’. Probably, the segments that have been defined are not static groups. It would be interesting to investigate how these groups develop in the course of time, which determinants influence this development, and whether it is possible to predict which young people will end up in which segment. With such knowledge, it would be possible to tune interventions to the target group even better.

In spite of the promising results, there are, of course, still some reservations concerning the investigation. Firstly, our study had a relatively low response. Despite two reminders and the incentive (the prospect of a €10 cinema ticket), somewhat fewer than half of the adolescents approached completed the questionnaire. However, the percentage of adolescents in this study who had recently consumed alcohol (48%) did not diverge from the data in the Brabant Youth Monitor (49% for the 12 to 18 age group). Within the Brabant Youth Monitor a larger number of young people had completed a questionnaire (N = 11,000). What is more, the response percentage in the Brabant Youth Monitor was also higher (57%). It therefore appears that in this investigation, at least in relation to alcohol consumption, we did not reach a very different group.

Secondly, this was a first explorative analysis. Validated questionnaires about values and attitude against alcohol for adolescents were at the time of our study not available. We therefore constructed our own questions, based on a literature study, expert opinions and focus groups of adolescents. With a factors analysis we found five different factors. However, it is recommended to confirm these factors in another sample of 12 to 18 years old. Although the segments were significantly associated with alcohol consumption variables independently of socio-demographic variables and the experts in the final workshop seemed to recognize these segments more research is needed to examine the usefulness of these different subgroups. Therefore, we were pleased that our 28 questions were added to the Brabant Youth Monitor 2011, a large questionnaire sent to more than 70,000 adolescents living in het province North-Brabant in order to strengthen the validation of our instrument.

Thirdly, it is not yet clear how we can identify and reach the different segments in everyday life. Furthermore, the results are still not conclusive about the segment that should be the target of policy. The size of the group suggests that we should concentrate primarily on the ‘ordinaries’, but if we consider the group where the most alcohol is consumed we should target the ‘high spirits’.

Because of these questions two new investigations have now been started in the Academic Collaborative Centre for Public Health Brabant. Grants for these investigations have been obtained from ZonMw, the Netherlands Organization for Health Research and Development. In a Ph.D. project the questions whether and how this audience segmentation (related to alcohol) and social marketing can improve the reach of both prevention and policy measures among adolescents aged 12 to 18 years will be studied. Moreover in this project we will examine whether existing effective interventions can be adapted, or whether new interventions can be developed that more closely match the specific characteristics, requirements, and needs of the different segments.

In addition, in another study we will investigate whether we can also distinguish different segments in the 16 to 24 age group.

## Conclusions

The results of this investigation demonstrate that it is possible to group adolescents into five different and more homogeneous segments on the basis of five attitude factors. Moreover, the five different segments also varied in drinking behavior independently of socio-demographic variables suggesting that they are meaningful alcohol related subgroups.

Our investigation was a first step in the search for possibilities of segmenting by other than socio-demographic characteristics. Further research is necessary in order to understand these results for alcohol prevention policy in concrete terms.

## Competing interests

The authors declare that they have no competing interests.

## Authors’ contributions

JM, was responsible for the data collection, the data analysis, and reporting the study results. All authors participated in the interpretation of the findings, reviewed the manuscript, and approved the final manuscript.

## Pre-publication history

The pre-publication history for this paper can be accessed here:

http://www.biomedcentral.com/1471-2458/12/742/prepub

## Supplementary Material

Additional file 1 Appendix Factor Loadings. Click here for file
