# Supplementary material for: Adolescents and alcohol: an explorative audience segmentation analysis
Source: BMC Public Health. 2012 Sep 5;12:742. doi: 10.1186/1471-2458-12-742 (PMC3490719; doi:10.1186/1471-2458-12-742)
Supplement: Additional file 1 — Appendix Factor Loadings. [file 1471-2458-12-742-S1.doc]

**Appendix Factor Loadings**

|  | **Aversion to intoxication** | **Alcohol as norm** | **Need for approval** | **Hedonistic associations** | **Lack of interest in alcohol** |
| --- | --- | --- | --- | --- | --- |
| I would be embarrassed if one of my friends got drunk | 0.77 | 0.11 | 0.00 | 0.00 | 0.04 |
| My friends would be embarrassed if I got drunk | 0.72 | 0.16 | 0.00 | -0.01 | 0.02 |
| I would be embarrassed if I got drunk myself | 0.69 | 0.00 | 0.03 | -0.06 | -0.07 |
| I would find it amusing if one of my friends got drunk | -0.56 | 0.23 | -0.06 | 0.08 | 0.02 |
| My parents would be embarrassed if I got drunk | 0.54 | 0.20 | -0.13 | -0.03 | 0.06 |
| People are more fun when they've been drinking | -0.48 | 0.22 | -0.06 | 0.25 | 0.10 |
| Stronger action should be taken against alcohol misuse | 0.48 | -0.15 | 0.03 | -0.02 | -0.16 |
| People become annoying when they've been drinking | 0.47 | -0.04 | 0.09 | -0.08 | -0.15 |
| I can imagine that you don't want to be seen with a soft drink when everyone else is drinking alcohol | 0.06 | 0.64 | 0.01 | 0.04 | 0.04 |
| It’s weird if an adult never drinks alcohol | 0.00 | 0.59 | -0.07 | 0.04 | 0.08 |
| Drinking alcohol is more fun when it's not allowed | -0.07 | 0.56 | -0.02 | 0.04 | 0.00 |
| Since it's legal to buy alcohol once you are 16, it must be less damaging from that age | 0.05 | 0.47 | -0.01 | 0.01 | -0.03 |
| I think it's exciting to be drunk | -0.39 | 0.46 | -0.04 | 0.07 | 0.00 |
| For me it's important not to be different from other people | -0.03 | 0.41 | 0.27 | -0.16 | 0.15 |
| Alcohol is more for boys than for girls | 0.17 | 0.40 | -0.04 | -0.02 | 0.18 |
| The opinion of my parents is important to me | 0.02 | 0.00 | 0.70 | -0.04 | 0.07 |
| My parents take my opinion seriously | -0.07 | -0.03 | 0.62 | -0.06 | -0.05 |
| It's important to me that my friends have a good opinion of me | -0.02 | 0.00 | 0.58 | 0.03 | 0.11 |
| It seems only natural to me to keep to my parents’ rules | 0.06 | -0.06 | 0.57 | -0.03 | 0.02 |
| I learn from my mistakes | -0.06 | -0.13 | 0.48 | 0.04 | -0.05 |
| Alcohol makes me think of the weekend | -0.17 | -0.04 | 0.07 | 0.69 | -0.08 |
| Alcohol makes me think of relaxing | -0.18 | 0.06 | -0.02 | 0.67 | -0.14 |
| Alcohol makes me think of having fun | -0.33 | 0.71 | 0.06 | 0.64 | -0.12 |
| Alcohol makes me think of a drink with a meal | 0.20 | -0.09 | -0.09 | 0.62 | 0.01 |
| Alcohol makes me think of letting go | -0.39 | 0.03 | 0.01 | 0.50 | 0.26 |
| Alcohol makes me think of adulthood | -0.14 | -0.22 | -0.04 | -0.48 | -0.22 |
| Alcohol makes me think: Don't like the taste | 0.29 | -0.02 | -0.08 | -0.24 | 0.59 |
| Alcohol makes me think: Not for me | 0.37 | -0.03 | -0.07 | -0.31 | 0.50 |
